# WMR-DepthwiseNet: A Wavelet Multi-Resolution Depthwise Separable Convolutional Neural Network for COVID-19 Diagnosis

**DOI:** 10.3390/diagnostics12030765

**Published:** 2022-03-21

**Authors:** Happy Nkanta Monday, Jianping Li, Grace Ugochi Nneji, Md Altab Hossin, Saifun Nahar, Jehoiada Jackson, Ijeoma Amuche Chikwendu

**Affiliations:** 1 School of Computer Science and Engineering, University of Electronic Science and Technology of China, Chengdu 611731, China; mh.nkanta@std.uestc.edu.cn; 2 School of Information and Software Engineering, University of Electronic Science and Technology of China, Chengdu 611731, China; ugochinneji@std.uestc.edu.cn (G.U.N.); kofijackson@uestc.edu.cn (J.J.); 3 School of Management and Economics, University of Electronic Science and Technology of China, Chengdu 611731, China; altabbd@uestc.edu.cn; 4 Department of Information System and Technology, University of Missouri-St. Louis, St. Louis, MO 63121, USA; snnnm@umsl.edu; 5 School of Information and Communication Engineering, University of Electronic Science and Technology of China, Chengdu 611731, China; ijeomaamuche@std.uestc.edu.cn

**Keywords:** Chest X-ray (CXR), Computed Tomography (CT), convolutional neural network, depthwise separable convolution, multiresolution analysis, wavelet

## Abstract

Timely discovery of COVID-19 could aid in formulating a suitable treatment plan for disease mitigation and containment decisions. The widely used COVID-19 test necessitates a regular method and has a low sensitivity value. Computed tomography and chest X-ray are also other methods utilized by numerous studies for detecting COVID-19. In this article, we propose a CNN called depthwise separable convolution network with wavelet multiresolution analysis module (WMR-DepthwiseNet) that is robust to automatically learn details from both spatialwise and channelwise for COVID-19 identification with a limited radiograph dataset, which is critical due to the rapid growth of COVID-19. This model utilizes an effective strategy to prevent loss of spatial details, which is a prevalent issue in traditional convolutional neural network, and second, the depthwise separable connectivity framework ensures reusability of feature maps by directly connecting previous layer to all subsequent layers for extracting feature representations from few datasets. We evaluate the proposed model by utilizing a public domain dataset of COVID-19 confirmed case and other pneumonia illness. The proposed method achieves 98.63% accuracy, 98.46% sensitivity, 97.99% specificity, and 98.69% precision on chest X-ray dataset, whereas using the computed tomography dataset, the model achieves 96.83% accuracy, 97.78% sensitivity, 96.22% specificity, and 97.02% precision. According to the results of our experiments, our model achieves up-to-date accuracy with only a few training cases available, which is useful for COVID-19 screening. This latest paradigm is expected to contribute significantly in the battle against COVID-19 and other life-threatening diseases.

## 1. Introduction

The global COVID-19 epidemic has infected 347 millions of people around the globe and over 5.5 million deaths confirmed by the World Health Organization (WHO) as of 25 January 2022 [1]. The main strategy for better managing this pandemic is to find, isolate, and care for patients as soon as possible. The ability to quickly, easily, affordably, and reliably identify COVID-19 pathology in a person is critical to abating the spread of COVID-19 contagion. The traditional method for detecting COVID-19 is actually reverse transcription polymerase chain reaction (RT-PCR) tests [2]. Small quantities of viral RNA are collected from a nasal swab, augmented, and evaluated during the RT-PCR test with virus detection signified generally with a fluorescent dye. Unfortunately, the RT-PCR procedure is time-consuming and manual, taking up to two days to complete. False-positive polymerase chain reaction (PCR) testing has also been recorded in some studies [3,4]. Imaging-technology-related techniques such as computed tomography (CT) imaging, CXR-imaging-based [5,6,7,8], and ultrasound imaging [9] are examples of other research methods. CT scanning machines are often troublesome to operate for COVID patients since they must often be moved to the CT room, the equipment must be thoroughly cleaned after each use, and there is a higher risk of radiation exposure [9].

CT has been successfully used as a supportive method for COVID-19 condition evaluation, despite the fact that it is not approved as a primal diagnostic means [6]. The most general and common CT findings are considered to be the ground-glass opacities (GGO), which is at the beginning stage, accelerating stage, and air space combination during the peak stage while the bronchovesicular congealing in the contusions and pulling bronchiectasis are both evident during the reception stage. Machine learning algorithms have been reported with significant performance for the diagnosis of COVID-19 using CXR and CT scans. Multilayered perceptron (MLP), as a common method of ANN, has shown promising prediction capability of COVID-19 cases with an acceptable accuracy [10].

The application of DL frameworks to diagnose COVID-19 from CT images has shown promise results in several studies [6,11,12]. CT scans and RT-PCR tests are relatively expensive [13], and clinicians are compelled to conduct limited testing for only vulnerable populations due to excessive demand. CXR imaging is a relatively low-cost form of detecting lung infections and it can also be used to detect COVID19 [14]. With relatively small and large datasets, convolutional neural networks (CNNs) have obtained up-to-date results in medical imaging research [15,16,17,18]. Due to the large number of parameters, CNNs can easily overfit on a small dataset; as a result, generalization efficiency is reciprocal to the dimension of the labeled data. Tiny datasets present the most difficult task in the medical imaging domain because of the restricted quantity and variety of samples [5,6,7].

A range of medical biomarkers and abnormalities have also been investigated as indicators of disease development in research, and there are some indications that imaging data could supplement these models [19,20,21]. While these methodologies have been utilized to examine COVID-19 in recent research, some have been employed to multi-institutional chest X-ray image samples [22,23]. The relationship between ground-glass obscurities and lung fusion on CXR with disease severity and development has been qualitatively characterized in recent research [24]. The study of attack detection and ECG-based biometric identification has utilized DL algorithm combined with wavelet multiresolution analysis extensively [25,26].

The aim of this research is to establish a conceptual depthwise separable convolution network with wavelet multiresolution analysis module for COVID-19 screening from chest X-ray (CXR) and computed tomography (CT). In consideration of a novel medical predicament such as COVID-19, obtaining adequately accessible compilation of medical image dataset for training deep learning (DL) algorithms is difficult due to the time and resources required to collect and mark images.

Medical image mining is a time-consuming and costly procedure that necessitates the involvement of radiologists and researchers [6]. Furthermore, due to the recent nature of the COVID-19 outbreak, adequate data of CXR images are difficult to come by. However, in COVID-19 AI-based screening systems from CT and CXR imaging, loss of spatial information is still a major concern which, in most occasions, results from the downsampling operation. The consequence of this is that the AI-based system will learn incomplete information from the data, thereby missing the distinct features for optimal classification.

In view to alleviate this drawback, we proposed a novel depthwise separable convolution network with wavelet multiresolution analysis module that optimizes the downsampling operation without losing spatial details for COVID-19 classification. The contributions of this work include: (1) Magnify the feature extraction robustness of the network by replacing the max-pooling layers with discrete wavelet transform (DWT) pooling for the loss reduction of spatial details and to achieve reduction in dimension without losing positional details by employing scaling and wavelet functions. (2) The depthwise separable connectivity framework ensures reusability of feature maps by directly connecting previous layer to all subsequent layers for extracting feature representations from few dataset. This enables the model to learn the spatial details for effective classification. (3) This paper is the first work that introduces a depthwise separable convolution network with wavelet multiresolution analysis module for feature extraction from radiograph images. The proposed model is an end-to-end learning techniques for COVID-19 classification that achieves much higher diagnosis accuracy.

The subsequent sections of this article is coordinated as follows. In Section 2, we survey related essays. In Section 3, we give a detailed explanation of the methodology, descriptive information about the dataset, and the implementation technicalities. The experimental outcomes are presented in Section 4. In Section 5, we give more light on the evaluation and validation of our model. In Section 6, we discuss the relevance of our proposed scheme. The concluding phase is detailed in Section 7.

## 2. Related Works

COVID-19 investigations based on DL algorithms have been on the rise in most research articles at present. The ImageNet weights were pretrained on 18-layer custom ResNet architecture against 100 COVID-19 and 1431 pneumonia instances of CXR dataset as proposed in [27].

According to Lu et al. [19], who adopted a neural network approach for the prediction of intensive care unit admission, concluded that biomarkers such as creatinine, C-reactive protein, etc. indicated momentary variations among admitted COVID-19 patients in the ward and transferred to the intensive care unit in contrast to the patients not transferred. Li et al. [28] formulated a DL model and a risk rating algorithm for the outcome of intensive care unit admission and death in the hospital. The ROC-AUC was utilized as a metric to evaluate the model performance. The authors discovered that these biomarkers were the leading ICU indicators, aside age, cardiac troponin, and oxygen saturation, which were the main death indicators. Similarly, Hou et al. [29] formulated a machine learning (ML) algorithm to predict the leading ICU admission and the main mortality indicators, which are temperature, procalcitonin, age, lactate dehydrogenase, lymphocytes, pulse oxygen saturation, ferritin, and C-creative protein.

Nneji et al. [30] suggested a scheme that combines wavelet transform and generative adversarial network (GAN) CNN in order to enhance the low quality of radiograph images for COVID-19 identification. A custom-based residual CNN approach was suggested in [31,32] to accurately differentiate COVID-19 instances from healthy CXR images and other pneumonia-related ailment. COVIDX-Net is a compilation of DL frameworks that were trained on 25 verified COVID-19 instances [33]. Recent studies have focused on automatic coronavirus pneumonia investigation from CT scans with encouraging results [34,35,36].

A ResNet-50 transfer learning based CNN algorithm was proposed in [37] to identify COVID-19 on a private dataset with an overall score of 94% accuracy against a regular standard CT slice. In [38], a weakly supervised approach was suggested where segmentation masks were produced automatically in which the CT image and mask are supplied to the algorithm for classification. The authors of this essay claimed that their procedure obtained 95.9% AUC. A combination of DL algorithm was suggest in [39] to achieve lung field segmentation by hybridizing 3D ResNet-50 transfer learning model with U-Net preprocessor model in a single architecture to classify COVID-19 and distinguish it from non-COVID-19 instances in a broad range of data from nonpublic datasets extracted from six hospitals. The authors of this study claim that their algorithm obtained 87.1% sensitivity.

ML approach was proposed in [40] to tackle the difficulty of automatically differentiating COVID-19 from other acquired pneumonia diseases. Infection-size-conscious techniques with random forest classifier algorithm was proposed in [41] to remove infection and lung areas by means of segmented scan to categorize images based on infection size using 1071 healthy and 182 COVID-19 instances. The authors of this essay claimed that the algorithm obtained 87.9% accuracy when trained on public and private datasets. A joint function pyramid network-based attention module and ResNet-50 proposed in [42] obtained 86.4% accuracy and 90.3% sensitivity when tested on a private dataset of 24 healthy and 27 COVID-19 individual instances. A DL-inspired random forest model was proposed in [43] to focus on extensive features to check COVID-19 severity. The procedure achieved an overall accuracy of 87.5% on 176 instances.

In summary, most studies, including those that have utilized CXR and CT imaging, rely on an insufficient number of COVID-19 images from various sources with no standardized protocols. They appear to be simple applications of existing AI-based algorithms leading to minimal AI innovation and clinical utility. The high data discrepancy associated with various studies causes comparison perplexity despite the fact that all models performed admirably [44]. Generally, models for COVID-19 examination and investigation based on CXR or CT images perform well.

Notwithstanding, a few models utilize10 COVID-19 test instances, and at least one model utilizes external validation attributed to data scarcity. As a consequence, they may or may not be applicable to other contexts. A system that uses less data and attains high significant achievement in accuracy with less training instances is required. This will permit to a greater extent the inclusion of uncommon data class in the testing set. The objective of this article is to formulate a scheme that can help to enhance previous models and achieve state-of-the-art results.

## 3. Materials and Methods

### 3.1. Datasets

**Figure 1:**
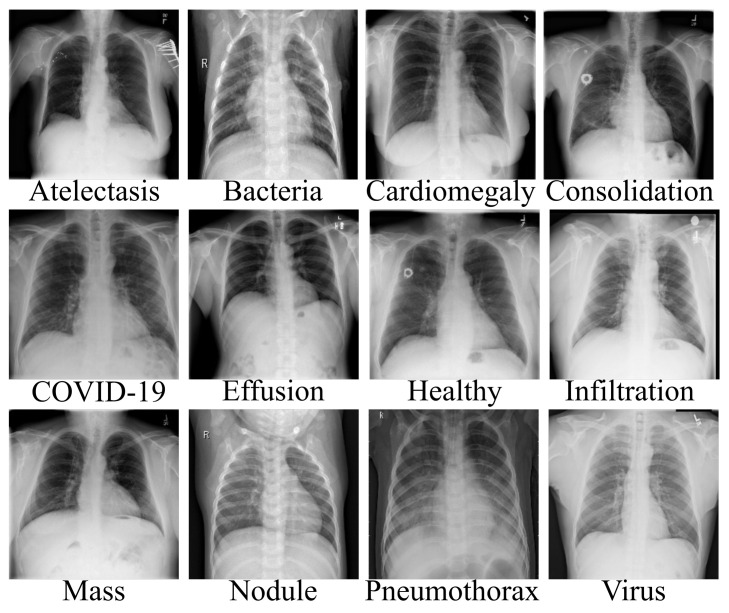
Data collection of chest X-ray images of different pneumonia-related illnesses including COVID-19.

**Figure 2:**
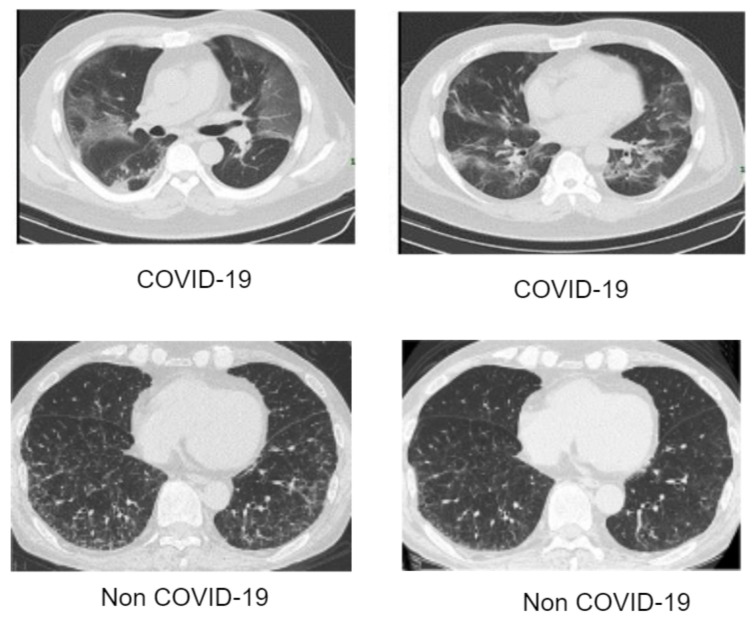
Data collection of computed tomography (CT) images of COVID-19 and non-COVID-19.

Artificial intelligence (AI) has achieved a remarkable reputation in the field of clinical research. In the face of the current pandemic ravaging our world, AI can assist healthcare workers in the process of disease detection, boosting the accuracy of identification methods at fast rate and perhaps saving lives. The scarcity of appropriate data is perhaps the most significant barrier facing AI-based approaches. Since AI-based approaches are data-driven, a large amount of data is needed. The process of data collection is quite tedious, as there are many ethics concerns from experts. Bearing this view in mind, we resorted to well-known and validated dataset repositories for the collection and compilation of the dataset. In this article, we collected CXR data of different pneumonia related illnesses from three different open sources [45,46,47,48]. As illustrated in Table 1, we collected 3029 scans of bacterial pneumonia, 8851 scans of healthy patients and 2983 scans of viral pneumonia from the Kaggle database of the Radiological Society of North America (RSNA) [45]. We collected 74,999 scans of other pneumonia-related illnesses from National Institute of Health (NIH) [46]. We collected 3616 scans of COVID-19 CXR from the COVID-19 radiography database [47] as illustrated in Table 1 for the purpose of validating our proposed architecture for multiple classification problems. The COVID-19 CT samples were obtained from COVID-19 dataset [48] as depicted in Table 2 for binary classification. As indicated, there are approximately 93,627 CXR scans including COVID-19 and 10 other pneumonia-related illnesses as well as healthy instances and a total of 2482 CT scans of COVID-19 and non-COVID-19 samples. Since the number of each category of data class varies, as a result, we selected 2000 scans of CXR from each category which sum up to 24,000 CXR images. Since the amount of CXR associated with each class is balanced, the dataset is partitioned into three sets of 70%, 20%, and 10% for training, validation, and test, respectively. Similarly, the CT dataset is also partitioned in the same manner from a selection of 1230 scans from each category. Figure 1 gives a visual representation of the dataset distribution for CXR scans while Figure 2 displays the visual representation for the CT scans.

### 3.2. Proposed WMR-DepthwiseNet

**Figure 3:**
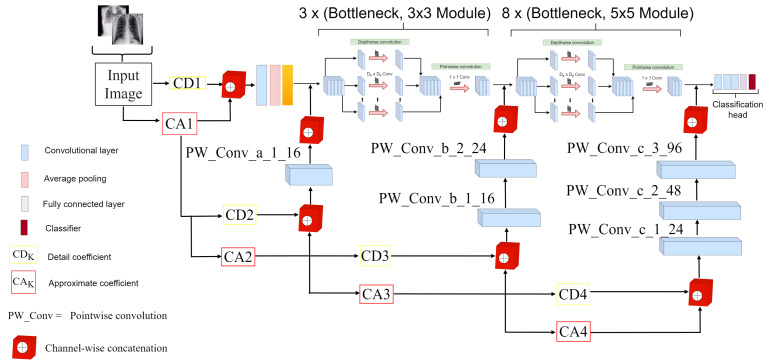
Overall structure of our proposed WMR-DepthwiseNet.

In this article, we proposed a deep convolutional neural network called the depthwise separable convolution network with wavelet multiresolution analysis module (WMR-DepthwiseNet) for the classification of COVID-19, healthy, and other pneumonia cases. As depicted in Figure 3, the core structure of WMR-DepthwiseNet is depthwise separable convolution connectivity and wavelet multiresolution analysis module. The parameters for the proposed depthwise separable convolution is presented in Table 3. To begin with, an initial 3×3 standard convolution is executed on the input radiograph image and the feature maps are fed as input into the depthwise separable convolution block to obtain features with the help of the depthwise separable convolution connectivity structure. The input features are concatenated with the output features by the depthwise separable convolution connectivity structure in an iterative manner that capacitates each convolution layer to receive raw details from all prior layers, which can achieve reusability of feature maps for the goal of extracting more features from fewer radiograph images.

Consequently, a pointwise convolution layer is introduced to accomplish subsampling. The pointwise convolution layer consists of 1×1 convolution, a rectified linear unit (ReLU), and a batch normalization (BN). A wavelet multiresolution analysis module is implemented to realize channelwise concatenation of both spatial and spectral details of the input and output feature maps to enable the network give more attention to positional details without loss of spatial information. The structure of the wavelet multiresolution analysis consists of 1×1 pointwise convolution, two coefficients of detail and approximate, and channelwise concatenation. At the tail end of the depthwise separable convolution block, a global average pooling is applied to the feature maps before sending them to the 1×1 convolutional layer instead of the conventional fully connected layer and then followed by a softmax layer for classifying the output of the prediction for the multiclass problem, whereas for the binary class problem, we substituted the softmax layer with sigmoid layer. To maintain a fix size of the feature maps, the padding is set to zero for all the convolution layers.

The overall strucure of the WMR-DepthwiseNet employs 3× bottleneck modules of 3×3 depthwise separable convolution, 8× bottleneck modules of 5×5 depthwise separable convolution, an efficient last stage of the classification head, and four levels of wavelet multi-resolution decomposition. The bottleneck module of the depthwise separable convolution and the wavelet multiresolution decomposition will be discussed in the following subsequent sections.

#### 3.2.1. Depthwise Separable Convolution Module

**Figure 4:**
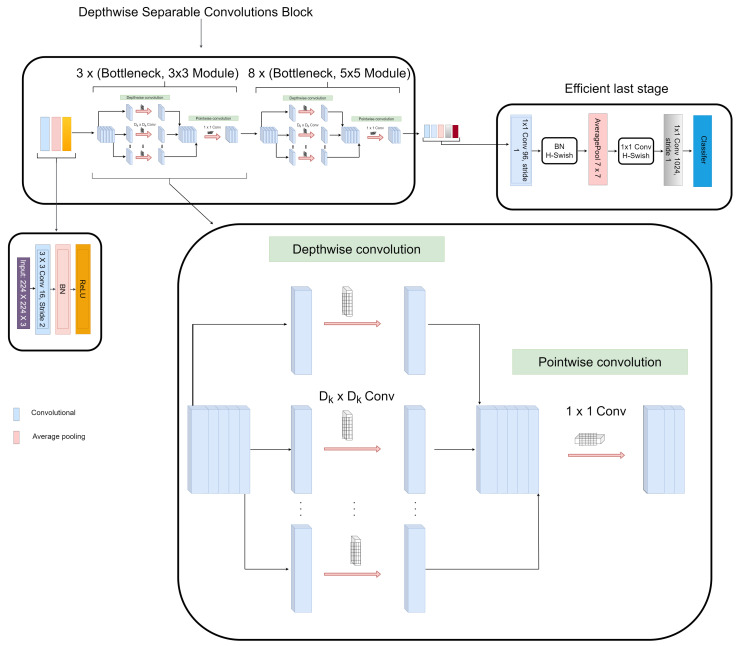
Detailed structure of our proposed depthwise separable convolution module.

**Figure 5:**
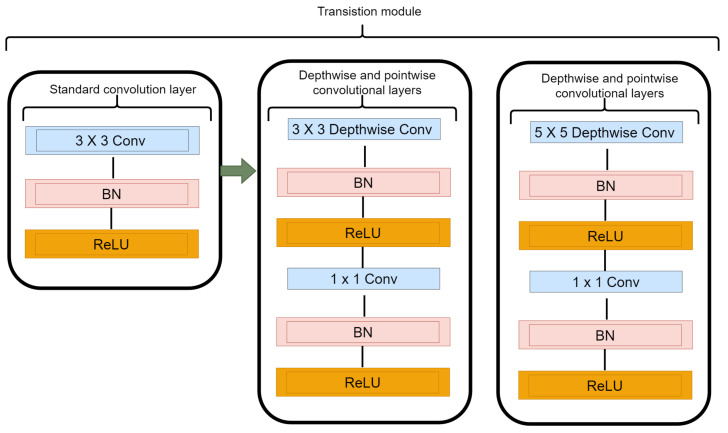
Transition of regular convolution to depthwise separable convolution module for both 3×3 and 5×5 depthwise convolutions.

Depthwise separable convolution module is a factorized form a conventional convolution that consists of depthwise convolution and a pointwise layer of 1×1 convolution. The depthwise convolution applies a single convolution filter for every input channel to carry out lightweight filtering operation. The pointwise layer of 1×1 convolution ensures that new features are created via computing simple summations of the input channels. The depthwise separable convolution layer depicted in Figure 4 while Figure 5 shows the transition from the regular convolution to depthwise separable convolution which is built with the following operations: 3×3 convolution, 5×5 convolution, batch normalization (BN), rectified linear unit (ReLU), 1×1 convolution, batch normalization (BN), and rectified linear unit (ReLU).

The 1×1 pointwise convolution is incorporated as a bottleneck layer to reduce the feature maps of the input before every 3×3 and 5×5 depthwise convolution, which enhances the computational efficiency. Since the number of feature map channels output by each depthwise separable convolution block contributes to the computational cost, the 1×1 pointwise convolution compresses the number of feature map channels of the input to be equivalent with the number of feature map channels of the output while the 3×3 depthwise convolution extracts details from the feature maps and ensures the number of the channels do not change.

The first depthwise separable convolutional module consists of three 3×3 bottleneck depthwise separable convolutional layers, and the second depthwise separable convolutional module consists of eight 5×5 bottleneck depthwise separable convolutional layers. Let $G_{d}\left(\cdot \right)$ represents depthwise separable convolution layers transformation, where *d* indexes the depthwise separable convolution layers and depicts the output of the *d*th depthwise separable convolution layers as $X_{d}$. For ensuring information flow enhancement between depthwise separable convolution layers within each depthwise separable convolution module, the depthwise separable convolution module uses direct connection from prior depthwise separable convolution layers to all subsequent depthwise separable convolution layers. That is, the *d*th depthwise separable convolution layer receives the feature maps of all the subsequent depthwise separable convolution layers, $Y_{0},.....,Y_{d-1}$ as depicted in Equation (1)

(1) $$Y_{d}=G_{d}\left(\left[Y_{0},Y_{1}...,Y_{d-1}\right]\right)$$

where $\left[Y_{0},Y_{1}...,Y_{d-1}\right]$ depicts the concatenation of the feature maps generated in the depthwise separable convolution layers 0,…,d−1. This type of depthwise separable connectivity framework realizes reusability of feature maps, which is capable of mining more features from the limited radiograph scans to enhance classification accuracy.

#### 3.2.2. Discrete Wavelet Transform

**Figure 6:**
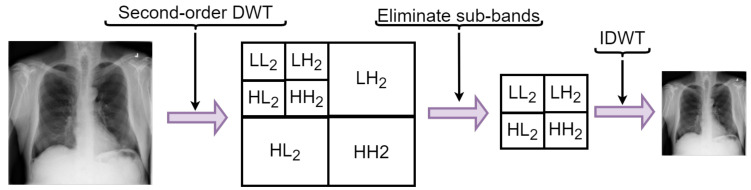
Illustration of discrete wavelet transform operation for downsampling.

To enhance the performance of identification without delineating infection lungs areas manually, a conventional approach is that if the model can unaidedly focus on infection areas without losing spatial information, the model would enhance its ability to differentiate the interclass discrepancies between COVID-19 and other pneumonia which will automatically enhance the classification accuracy. Conventional CNNs mainly adopts pooling layers for downsampling operation to achieve reduction in dimensionality, which usually lead to loss of spatial information. Some advanced CNNs have adopted channel and spatial attention mechanisms to strengthen the network’s critical information adaptability for aggregating and unaidedly recalibrating feature maps through spatialwise and channelwise approaches. Several attention schemes have been proposed such as squeeze-and-excitation [49], bottleneck attention scheme [50], SCA-CNN [51], and so on. The major drawbacks of the attention schemes is that it sometimes lead to decline in performance and accuracy when the feature map is multiplied with two attention maps and the weight map produced during the early phase of the network’s training when the parameters are not well trained. To alleviate the above drawbacks, we introduce discrete wavelet transform pooling to replace the conventional pooling operation in standard CNNs, which enables the network to retain spatial information and ensure dimensionality reduction without loss of information and spatial details thereby improving the network performance and computational efficiency. Let ψ(·) denote the wavelet function defined over the main axis (−∞,∞) where the integral of ψ(·) is zero as presented in Equation (2). Figure 6 shows the operation of discrete wavelet transform for downsizing.

(2) $$\int_{-\infty }^{\infty }\psi \left(u\right)du=0$$

The integral of the square of ψ(·) is unity as presented in Equation (3)

(3) $$\int_{-\infty }^{\infty }\psi^{2}\left(u\right)du=1$$

Equation (4) explicitly expresses the admissibility condition.

(4) $$C_{\psi }=\int_{0}^{\infty }\frac{{\left|\psi \left(f\right)\right|}^{2}}{f}dfsatisfies0<C_{\psi }<\infty$$

By converting and stretching this mother wavelet as shown in Equation (5), a twofold-indexed family of wavelets can be formed.

(5) $$\psi_{\lambda ,t}\left(u\right)=\frac{1}{\sqrt{\lambda }}\psi \left(\frac{u-t}{\lambda }\right)$$

where λ>0 and *t* is 1, the normalization on the right hand side of Equation (5) is chosen such that $\left|\right|\psi_{\lambda ,t}\left|\right|=\left||\psi |\right|$ for all λ, *t* and $\frac{1}{\sqrt{\lambda }}$ is the normalizing term.

#### 3.2.3. Wavelet Multiresolution Analysis

**Figure 7:**
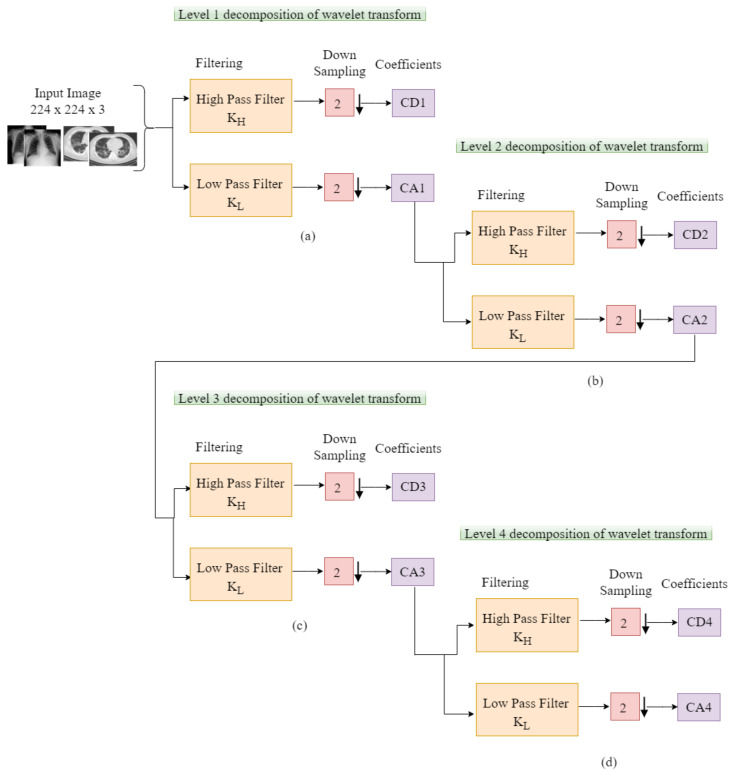
(**a**)–(**d**) Detailed structure of the wavelet multiresolution analysis of four-level decomposition.

Our proposed scheme allows us to connect depthwise separable convolution with wavelet multiresolution analysis to achieve filtering and downsampling. The wavelet multiresolution analysis algorithm formulates pointwise convolution and pooling into depthwise separable convolutional neural network as filtering and downsizing. The proposed scheme executes pooling operation to pool features by carrying out four-level decomposition of two-dimensional discrete wavelet transform as depicted in Figure 7. The structure of the wavelet multiresolution analysis consists of two filter banks of high and low pass filters, scaling factor of 2 for downsampling operation, and the generated detail and approximate components. Equations (6) and (7) depict the multiresolution analysis of the wavelet transform for both the scaling and wavelet functions, respectively.

(6) $$W_{\psi }\left(k+1,m\right)=h_{\psi }\left(-j\right)*W_{\psi }\left(k,j\right){|}_{j=2m,m\leq 0}$$

(7) $$W_{\Psi }\left(k+1,m\right)=h_{\Psi }\left(-j\right)*W_{\Psi }\left(k,j\right){|}_{j=2m,m\leq 0}$$

where $W_{\psi }$, $W_{\Psi }$ are the approximate and detail components, respectively. The approximate function is denoted by ψ and ψ represents the detail function. $h_{\psi }\left(-n\right)$ represents the time inverse scaling while the wavelet distributions is denoted by $h_{\Psi }\left(-j\right)$. The variable in the distribution is denoted as (n), whereas the resolution scale is depicted as (k). First, the discrete wavelet transform (DWT) is applied on the rows, and second, it is applied on the columns to achieve detail and approximate sub-bands. LH, HL, and HH are the sub-bands of the detail component at every level of decomposition while LL is the approximate sub-band at the highest order of the decomposition analysis.

Subsequently, the fourth-order sub-band is used to recapture the image characteristics after conducting the fourth-order decomposition. With the utilization of the inverse WT, which is based on the inverse DWT (IDWT), the image characteristics is pooled by a factor of 2 as depicted in Equation (8)

(8) $$W_{\psi }\left(k,m\right)=h_{\psi }\left(-j\right)*W_{\psi }\left(k+1,j\right)+h_{\Psi }\left(-j\right)*W_{\Psi }\left(k+1,j\right){|}_{j=2m,m\leq 0}$$

## 4. Results

### 4.1. Experimental Details

The WMR-DepthwiseNet is composed of two segments; the first segment is the depthwise separable convolution block with each layer of depthwise separable convolution module densely connected. The second segment is the wavelet multiresolution analysis module connected to the depthwise separable convolution block with concatenation channel connection to link subsequent layer with all the prior layers in order to avoid the loss of information about the input and positional details as the information moves along the network. Many state-of-the-art models have adopted similar technique such as ResNet and residual models and achieved good results in several machine vision problems, but a great amount of computational resources is needed due to the huge amount of parameters.

WMR-DepthwiseNet processes the input image through depthwise separable convolution layers. Precisely, the multiresolution analysis decomposes the input image via the low-pass and high-pass filters and concatenate the decomposed images into the depthwise separable convolution block channel-wise. The connection channels are executed using 1×1 pointwise convolutions. Finally, global average pooling is utilized to obtain a vectorized feature of the final output of the depthwise separable convolution block before feeding it to the 1×1 convolution followed by the classifier for identification. In this work, we utilized 1×1 convolution instead of the conventional fully connected layer.

### 4.2. Experimental Setup

To investigate the performance of our proposed model on screening COVID-19, we collected public dataset of both CXR and CT images from three open sources [45,46,47,48]. Since it is challenging to collect the different pneumonia-related illnesses from one data source especially COVID-19 cases, we put a dataset together from different open sources.

However, viral pneumonia dataset consist of 2983 scans of CXR, which is relatively the smallest when compared to the other CXR categories. As a result, we selected 2000 scans from each category for this study, bringing to a total of 24,000 CXR images as presented in Table 1. Since the amount of CXR linked with each class is balanced, the dataset is split into three portions. The training partition has 70% scans, the validation partition has 20% scans, and the test partition has 10% scans. In a similar manner, 1230 CT scans were selected to form a balance class with the same split ratio as presented in Table 2. During the process of feature extraction, the model is trained on the train dataset of 70% and validated simultaneously on the validation dataset of 20%. The remaining 10% of the dataset is used to test the model’s performance.

We utilized a dropout of 0.5 to avoid overfitting. An Adam optimizer with a learning rate of $1\times 10^{4}$ is used to train the proposed model for 30 epochs with batch size of 32. We trained our proposed WMR-DepthwiseNet on NVIDIA GTX1080. Keras is used for the construction of the WMR-DepthwiseNet scheme. The loss function utilized in this work is cross-entropy presented in Equations (9) and (10).

(9) $$CE_{loss}=-\sum_{i=1}^{12}yi\;log\left(pi\right)$$

where *i* denote the distribution of the class which is 12 categories, yi denote the class label, and pi is the predicted class.

(10) $$CE_{loss}=-\sum_{i=1}^{2}yi\;log\left(pi\right)$$

where *i* denote the distribution of the class which is 2 categories, yi denote the class label, and pi is the predicted class.

## 5. Evaluation

In this section, we presents an ablation study of the structural configuration of our proposed model with different depthwise bottleneck modules. We selected a few pretrained models and compared them with our proposed network in terms of classification performance using the same dataset. we only fine-tuned the last layer to correspond to the number of classes in our dataset. Another study was conducted to compare our proposed network with several state-of-the-art COVID-19 imaged-based screening methods.

In order to verify the effectiveness of our proposed model, we compared our designed WMR-DepthwiseNet with up-to-date models. For fair comparison, we run four state-of-the-art COVID-19 methods on the same dataset. From all indications, our proposed model outperforms the up-to-date methods and the deep learning pretrained models with a promising performance. The evaluation criterion adopted as the metric to evaluate the diagnosis performance of our proposed WMR-DepthwiseNet is as follows: accuracy (ACC), precision (PRE), sensitivity (SEN), specificity (SPE), area under curve (AUC), and F1-Score.

(11) $$F_{1}=2\times \frac{Precision\times Recall}{Precision+Recall}$$

(12) $$Accuracy=\frac{TP+TN}{TP+TN+FP+FN}$$

(13) $$Sensitivity=\frac{TP}{TP+FN}$$

(14) $$Specificity=\frac{TN}{TN+FP}$$

where TP, FP, and FN indicates the outcomes of true positive, false positive, and false negative, respectively.

### 5.1. Ablation Study

Firstly, we performed ablation study on different configurations of our proposed WMR-DepthwiseNet with different depthwise separable convolution bottlenecks. Particularly, we made comparison on the following architectures.

- WMR-DepthwiseNet-A: 3× (bn 3 × 3) + 5× (bn 5 × 5): This network employs 3× bottleneck modules of 3×3 depthwise separable convolution, 5× bottleneck modules of 5×5 depthwise separable convolution.
- WMR-DepthwiseNet-B: 3× (bn 3 × 3) + 6× (bn 5 × 5): This network employs 3× bottleneck modules of 3×3 depthwise separable convolution, 6× bottleneck modules of 5×5 depthwise separable convolution.
- WMR-DepthwiseNet-C: 3× (bn 3 × 3) + 7× (bn 5 × 5):This network employs 3× bottleneck modules of 3×3 depthwise separable convolution, 7× bottleneck modules of 5×5 depthwise separable convolution.
- WMR-DepthwiseNet-D: 3× (bn 3 × 3) + 8× (bn 5 × 5):This network employs 3× bottleneck modules of 3×3 depthwise separable convolution, 8× bottleneck modules of 5×5 depthwise separable convolution.

The experimental results of the ablation study are summarized in Table 4 and Table 5. At first, we evaluated our proposed network using the same dataset to examine the effect of different depthwise saparable bottleneck modules on the performance of the models. The number of 5×5 depthwise separable convolution bottleneck utilized varied from 5×, 6×, 7×, and 8×.

**Figure 8:**
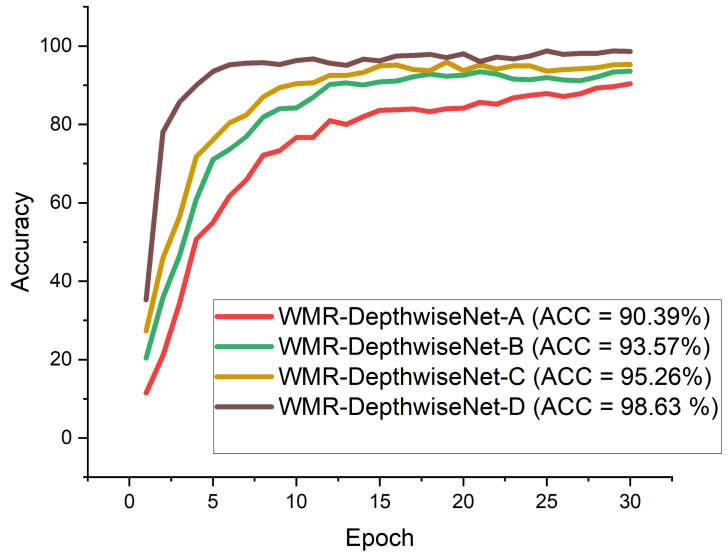
Accuracy curves showing the performance of our proposed WMR-DepthwiseNet in comparison with some selected up-to-date COVID-19 models using the same CXR dataset.

**Figure 9:**
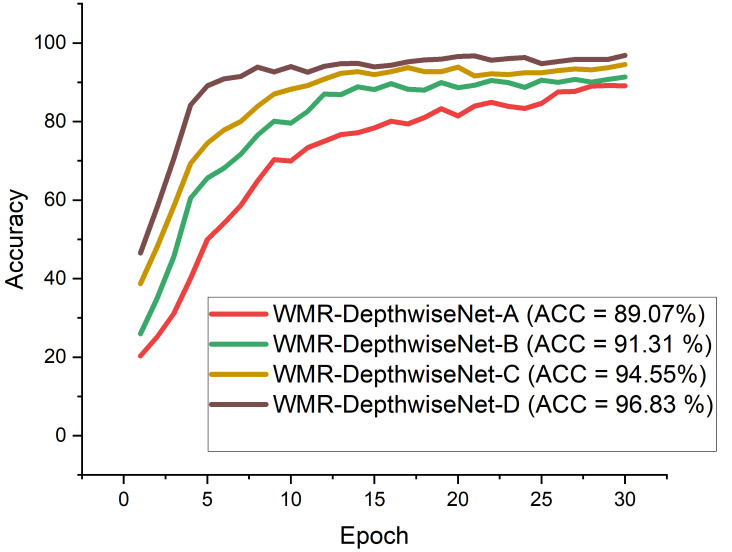
Accuracy curves showing the performance of the formulated WMR-DepthwiseNet in comparison with some selected up-to-date COVID-19 models using the same CT dataset.

From all indications, the WMR-DepthwiseNet-D with 3×(bn3×3) + 8×(bn5×5) achieved the highest performance across all the metrics. WMR-DepthwiseNet-A with 3×(bn3×3) + 5×(bn5×5) achieve the least score of 92.71% sensitivity as presented in Table 4 on CXR dataset and 91.46% sensitivity on CT dataset as presented in Table 5. However, an average increment of 4.38% was achieved on both CXR and CT dataset when a depthwise saparable bottleneck modules of 8×(bn5×5) is adopted as shown in Table 4 and Table 5. It is worth mentioning that the WMR-DepthwiseNet-D with 3×(bn3×3) + 8×(bn5×5) preserve more spatial details and hence improves model performance. To this end, our combined depthwise separable convolution network with wavelet multiresolution analysis module called WMR-DepthwiseNet-D achieves the best result across all evaluation metrices using both CXR and CT dataset as represented in Figure 8 and Figure 9. More to the point, the strategy of combining depthwise separable convolution network with wavelet multiresolution analysis enhances the performance of the WMR-DepthwiseNet by a wide margin.

### 5.2. COVID-19 Classification Evaluation

We compare the findings of our proposed model with well-known CNN pre-trained models and up-to-date COVID-19 screening methods. According to Table 6 and Table 7, our proposed WMR-DepthwiseNet outperforms all the selected pretrained models yielding state-of-the-art results using the same CXR and CT dataset. Our proposed approach yields 98.46% sensitivity, 97.99% specificity, 98.63% accuracy, 98.72% AUC, 98.87% precision, and 98.92% F1-score on CXR dataset as shown in Table 6. Table 7 shows that our model yields 97.78% sensitivity, 96.22% specificity, 96.83% accuracy, 97.61% AUC, 97.02% precision, and 97.37% F1-score on CT dataset.

**Figure 10:**
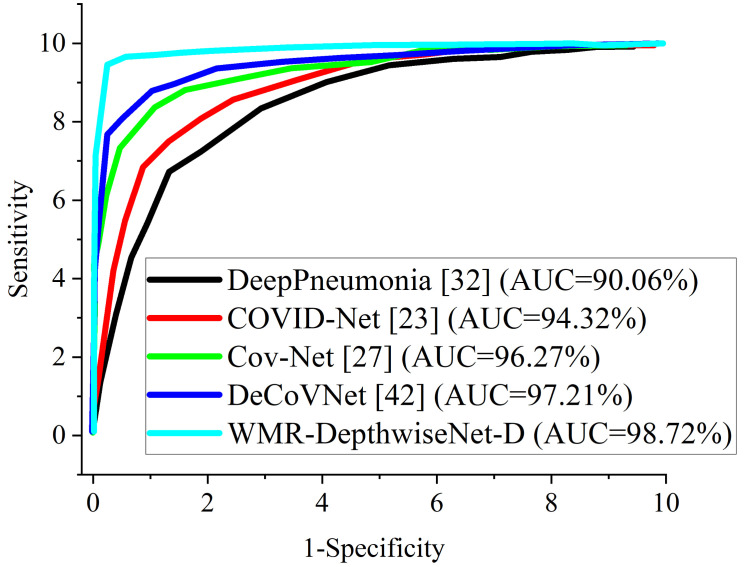
ROC-AUC curves of our proposed WMR-DepthwiseNet in comparison with some selected up-to-date models using the same CXR dataset.

**Figure 11:**
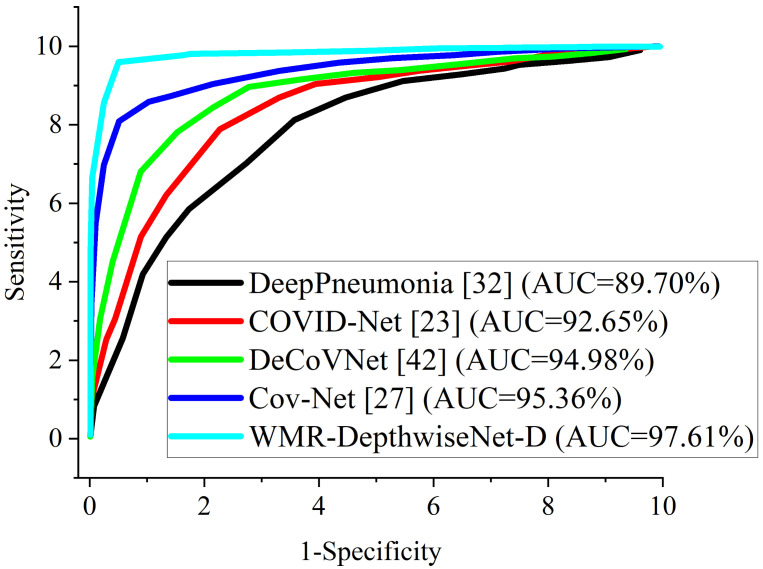
ROC-AUC curves of our proposed WMR-DepthwiseNet in comparison with some selected up-to-date models using the same CT dataset.

**Figure 12:**
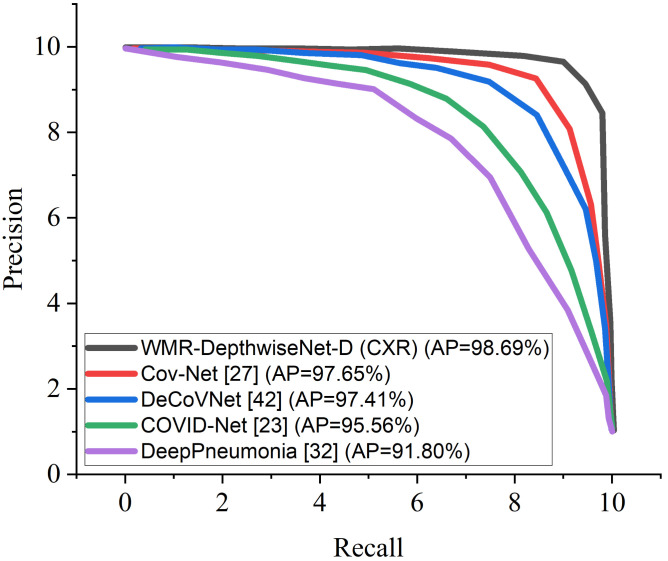
Precision-Recall curves of the formulated WMR-DepthwiseNet in comparison with some selected up-to-date models using the same CXR dataset.

**Figure 13:**
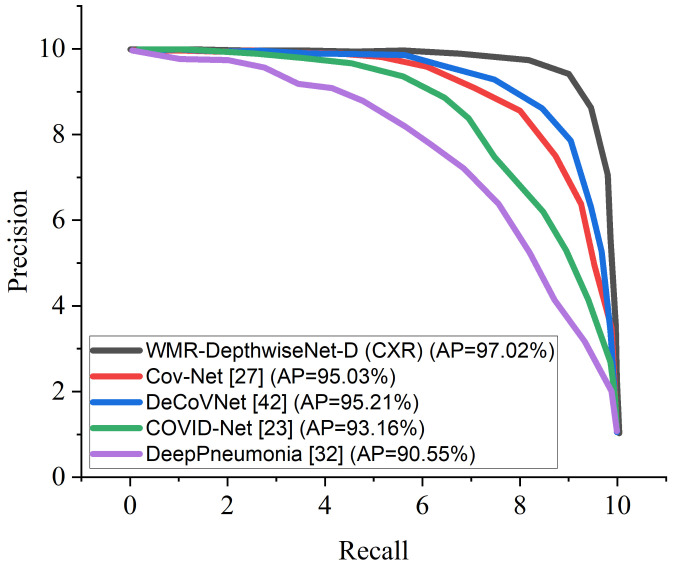
Precision-Recall curves of the formulated WMR-DepthwiseNet in comparison with some selected up-to-date models using the same CT dataset.

Figure 8 and Figure 9 illustrate the stability and convergence of the proposed WMR-DepthwiseNet-D in the test curve of the accuracy graphs for both CXR and CT datasets, respectively. More so, the performance of our proposed model can also be seen in the ROC-AUC curve as illustrated in Figure 10 and Figure 11 for both CXR and CT dataset respectively. The precision–recall curve is another important performance metric we adopted in our comparison as presented in Figure 12 and Figure 13. From all indications, our proposed model outweighs all the other models across all the evaluation metrics. Owing to our depthwise separable convolution network with wavelet multiresolution analysis module, the model achieves 98.63% accuracy on CXR dataset and 96.83% accuracy on CT dataset. The efficacy of integrating wavelet multiresolution analysis module with depthwise separable convolution network to modify the learning process is demonstrated by this result. Our model achieves 98.72% AUC, which is significantly higher than the other approaches. These findings confirm the benefits of wavelet multiresolution analysis module in our proposed model. Our proposed approach also achieves the highest specificity score of 97.99%, demonstrating the critical function of the WMR-DepthwiseNet.

As several attempts at COVID-19 classification have been made, we are now comparing the findings of our proposed WMR-DepthwiseNet with previous up-to-date COVID-19 screening methods. In detecting COVID-19 from CT exams, Chen et al. [11] uses CNN-based U-Net++ to recapture attributes from high-resolution CT exams for detecting COVID-19. The authors reported an accuracy of 95.2%. Shi et al. [41] uses an infection-size-based random forest approach to obtain region-specific attributes from CT exams for COVID-19 classification achieving 89.4% accuracy.

In separating COVID-19 from other viral pneumonia, Xu et al. [52] and Wang et al. [33] works are quite impressive achieving overall accuracy of 86.7% and 82.9%, respectively. However, their biggest flaw was that they only calculated a few indicators, which was insufficient to adequately represent the classification’s overall results. Song et al. [42] formulated a deep learning scheme called deepPneumonia to distinguish COVID-19 instances using CT exams achieving 86.1% accuracy. Using CT scans, Wang et al. [12] detected COVID-19 using CNN achieving 92.36% accuracy. Jin et al. [53] utilized a logistic regression scheme for detecting COVID-19. The authors claimed that their method achieved 96.5% accuracy. Jin et al. [54] formulated an AI-based scheme for detecting COVID-19, achieving 95.7% accuracy. Barstugan et al. [40] formulated a ML scheme for classifying COVID-19 using CT scans and achieved 90.7% accuracy. Table 8 presents a summary of the aforementioned methods in comparison with our proposed scheme. Tabrizchi et al. [55] suggested an enhanced densely connected convolutional networks (DenseNet) technique for three class classification based on transfer learning (TL). The proposed model obtained an overall accuracy of 95.7%, sensitivity of 87.4%, and specificity of 95.7%. The authors claimed that the high performance of their suggested TL model is due to the classifier’s robustness in dealing with imbalanced data classes and fewer datasets.Tabrizchi et al. [56] conducted a review of previously published methods and used artificial intelligence (AI) image-based diagnosis methods to detect coronavirus infection with zero or near-zero false positives and false negatives. The goal of their research is to develop the best accurate COVID-19 detection method among AI approaches including machine learning (ML), artificial neural networks (ANN), and ensemble learning (EL). The machine learning model with SVM classifier surpasses the order models, with accuracy of 99.2%, precision of 98.2%, and recall of 100%.

In another experiment, we compared the formulated WMR-DepthwiseNet with four selected COVID-19 models using the same dataset for fairness. Cov-Net [37] and DeCoVNet [53] show quite an impressive result followed by COVID-Net [33]. However, our proposed model outperforms the aforementioned COVID-19 models including DeepPneumonia [42], which had previously yielded up-to-date results and the other models as dipicted in Table 9 and Table 10 using the same CXR and CT dataset. Though the complex lung structures and indistinct infection areas pose unusual challenges, our proposed framework still achieves accurate results, demonstrating its robust strengths.

The proposed WMR-DepthwiseNet has competitive classification efficiency for COVID-19 recognition. The underlying explanation may be that the proposed WMR-DepthwiseNet can better utilize the extracted features of high-level discriminative representation. It is worth noting that the the deptwise separable convolution network with wavelet multiresolution analysis module can handle small-scale data while using less computing power than conventional deep-learning-based approaches. To further examine the performance of the suggested scheme with different hyper-parameter tuning, we presented a statistical report in Table 11 showing the yielded results by the formulated scheme using CXR and CT datasets. with a learning rate of 0.1 and 25% dropout using SGD optimize, the model obtained the least score of 88.18% accuracy on the CXR dataset. For the CT dataset, the model obtained the least score of 89.33% accuracy using RSMprop optimizer with a larning rate of 0.01 and 25% dropout. Utilizing 0.50 dropout and learning rate of 0.0001, the model obtained the best accuracy score of 98.63% and 96.83% with Adam optimizer on both CXR and CT datasets, respectively.

### 5.3. Cross-Dataset Evaluation

Despite the outstanding results record by the proposed model, we also presented cross-dataset evaluation to investigate if there is any discrepancy regarding the results obtained. We study the influence of training a model in one data distribution and evaluating it in another in this experiment. This situation is more realistic because training a model with images from all available sensors, environments, and persons is nearly impossible. We maintain same manner of data split of training, validation and test with 50%, 25%, and 25%, respectively, as shown in Table 12 and Table 13. The COVID-CXR scans dataset [47] is utilized for training the system while the COVID-CXR scans dataset [58] is utilized for the testing. Similarly, for the CT dataset, The COVID-CT scans dataset [48] is utilized for training the system while the COVID-CT scans dataset [59] is utilized for the testing. We ensured that no images from the training dataset source are present in the test dataset source. We adopted the well-known dataset reported in [47,48,58,59] for the experiment because it has been used by various researchers in the literature.

Although we emphasize that the training images used to train the model and the test images are drawn from different distributions. Other test designs were also investigated, such as employing the COVID-CXR training partition as a test and combining both COVID-CXR partitions as a bigger test set (See Table 12). We employed similar approach to the COVID-CT dataset (See Table 13). We also examine the inverse scenario in which the train and test set from the COVID-CXR dataset [58] are used for training and the train set from the COVID-CXR dataset [47] are used for testing. Similarly, the train and test set from the COVID-CT dataset [48] are used for training and the train set from the COVID-CT dataset [59] are used for testing.

When we examine cross-dataset assessment to intra-dataset evaluation, the model performance did not change that much which indicates that there is no significant bias in the results reported using the intra-dataset. Table 14 presents the results for the cross-dataset evaluation on the proposed model for the CXR dataset while Table 15 presents the results for the CT dataset. We believe that the slight changes in the performance of our model can be attributed to the data acquisition variation. Images from distinct dataset can be taken using different equipment and image sensors, causing relevant features on the images to change, yet the proposed model performed satisfactorily.

## 6. Discussion

It is important to make some remark about the proposed depthwise separable convolution network with wavelet multiresolution analysis module. Manual detection of COVID-19 by an expert utilizing CXR and CT can have a high sensitivity but a low specificity of 25%. This inadequate specificity leads to false positive predictions, which leads to ineffective therapy and wasted money. Our proposed WMR-DepthwiseNet has a high specificity of 96.22%, which can be used to help expert radiologists reduce the number of false positive instances reported.

More importantly, the stated result in terms of Receiver Operating Characteristic (ROC) can aid expert radiologist in achieving a trade-off between specificity and sensitivity by telling the overall accuracy as illustrated in Figure 10 and Figure 11. The Receiver Operating Characteristic (ROC) maximizes the true positive prediction and also minimizes the false positive rate. From the ROC curve, it is obvious that the formulated model outperforms the other algorithms with the overall accuracy of 98.72% on CXR dataset and 97.61% on CT dataset.

More interestingly, the precision–recall curve also shows that our proposed WMR-DepthwiseNet outweighs the other models with an average precision of 98.69% on CXR dataset and 97.02% on CT dataset. The precision-recall graph demonstrates the trade-off between precision and sensitivity. It is obvious that the model performs better than the other up-to-date COVID-19 models as shown in Figure 12 and Figure 13 which means our model has higher precision associated with higher sensitivity.

Furthermore, some comments on WMR-DepthwiseNet computational cost and model complexity are necessary. We combined depthwise separable convolution network with wavelet multi-resolution analysis module for feature extraction. We adopted wavelet pooling instead of the usual max-pooling operator for down-sizing operation which reduced model complexity and computation time. Another intriguing feature of our WMR-DepthwiseNet is its capacity to preserve high-level features without loss of spatial details. In terms of computing cost, the formulated algorithm was trained on an NVIDIA GTX 1080 and implementation on Keras framework. In comparison to earlier up-to-date models, the complexity of the proposed scheme is much reduced with fewer parameters as a result of the wavelet pooling strategy adopted. In all the assessment metrics, the proposed WMR-DepthwiseNet outperforms their counterparts as depicted in Table 8. Our proposed strategy consistently produces better performance in terms of SEN, SEP, ACC, AUC, PRE, and F1Score. The explanation for this is that our proposed WMR-DepthwiseNet learns high-level discriminative details. Furthermore, the WMR-DepthwiseNet outperforms up-to-date approaches with better classification results.

## 7. Conclusions

We propose a CNN called depthwise separable convolution network with wavelet multiresolution analysis module (WMR-DepthwiseNet) in this paper with the objective of addressing the issue of low performance in COVID-19 screening from radiograph (CXR and CT) images as well as loss of spatial details during feature extraction. We implemented a depthwise separable convolution network with wavelet multi-resolution analysis and a discrete wavelet transform (DWT) pooling to replace the conventional max-pooling operation as a strategy to avoid loss of spatial details and to preserve high-level feature and learn the distinctive representations for COVID-19 classification. We have demonstrated that our proposed model is effective and converges very fast with better classification performance. By a broad margin, our proposed approach outshone previous up-to-date COVID-19 diagnostic strategies. The limitation of our work is that we did not consider imbalance problem which happens to be the case for newly discovered diseases due to lack of sufficient data which usually leads to uneven class distribution. In our future work, we will also focus on imbalance class problem. We belief that graph-based convolutional neural network can improve the quality of the result. Therefore, part of our future work will take into consideration the possibility of implementing graph depthwise separable convolutional network.

## Figures and Tables

**Table 1:** Description of the chest X-ray (CXR) dataset showing different categories of pneumonia illnesses and the distribution of images per category as well as the number of selected images per category.

Dataset	Category of Pneumonia	Data Count per Category	Selected No. of Data Category	Training Set	Validation Set	Test Set
RSNA [45]	Bacteria	3029	2000	1400	400	200
	Viral	2983	2000	1400	400	200
	Healthy	8851	2000	1400	400	200
NIH [46]	Atelectasis	4999	2000	1400	400	200
	Cardiomegaly	10,000	2000	1400	400	200
	Consolidation	10,000	2000	1400	400	200
	Effusion	10,000	2000	1400	400	200
	Infiltration	10,000	2000	1400	400	200
	Mass	10,000	2000	1400	400	200
	Nodule	10,000	2000	1400	400	200
	Pneumothorax	10,000	2000	1400	400	200
Rahman et al. [47]	COVID-19	3616	2000	1400	400	200
	Total	93,627	24,000	16,800	4800	2400

**Table 2:** Description of the computed tomography (CT) dataset showing COVID-19 and non-COVID-19 categories including the distribution of images per category as well as the number of selected images per category.

Dataset	Category of Pneumonia	Data Count per Category	Selected No. of Data Category	Training Set	Validation Set	Test Set
Silva et al. [48]	COVID-CT	1252	1230	861	246	123
	NON-COVID-CT	1230	1230	861	246	123
Total		2482	2460	1722	492	246

**Table 3:** Parameter for the proposed depthwise separable convolution. bnk represents bottleneck convolution. NLT stands for the kind of nonlinearity adopted. HSW represents h-swish. REL denotes ReLU and SD represents stride.

Input	Operator	Expansion Size	Output	NLT	SD
224 × 224 × 3	Conv2d, 3×3	-	16	HSW	2
112 × 112 × 16	bnk, 3×3	16	16	REL	2
56 × 56 × 16	bnk, 3×3	72	24	REL	2
28 × 28 × 24	bnk, 3×3	86	24	RE	1
28 × 28 × 24	bnk, 5×5	96	40	HSW	2
14 × 14 × 40	bnk, 5×5	240	40	HSW	1
14 × 14 × 40	bnk, 5×5	240	40	HSW	1
14 × 14 × 40	bnk, 5×5	120	48	HSW	1
14 × 14 × 48	bnk, 5×5	144	48	HSW	1
7 × 7 × 96	bnk, 5×5	288	96	HSW	2
7 × 7 × 96	bnk, 5×5	576	96	HS	1
7 × 7 × 96	bnk, 5×5	576	96	HSW	1
7 × 7 × 256	Conv2d, 1×1	-	256	HSW	1
1 × 1 × 256	Avg pool 7×7	-	-	-	1
1 × 1 × 1024	Conv2d, 1×1	-	1024	HSW	1

**Table 4:** Performance evaluation of the structural configuration of our proposed model with different depthwise bottleneck modules for CXR dataset. bn represents the bottleneck module.

Structural Models	SEN (%)	SPE (%)	ACC (%)	AUC (%)	PRE (%)	F1-Score (%)	Time (min)
WMR-DepthwiseNet-A: 3 (bn 3×3) + 5 (bn 5×5)	92.71	91.84	90.39	91.14	91.67	92.12	13.5
WMR-DepthwiseNet-B: 3 (bn 3×3) + 6 (bn 5×5)	97.5	96.22	93.57	96.93	95.42	96.15	14.2
WMR-DepthwiseNet-C: 3 (bn 3×3) + 7 (bn 5×5)	98.17	97.85	95.26	97.11	96.64	97.3	14.9
WMR-DepthwiseNet-D: 3 (bn 3×3) + 8 (bn 5×5)	98.46	97.99	98.63	98.72	98.69	98.92	15.6
WMR-DepthwiseNet-D: 3 (bn 3×3) + 9 (bn 5×5)	96.3	94.6	95.6	94.8	96.2	95.1	16.7
WMR-DepthwiseNet-D: 3 (bn 3×3) + 10 (bn 5×5)	95.2	94.4	93.7	94.1	95.8	96.8	17.1

**Table 5:** Performance evaluation of the structural configuration of our proposed model with different depthwise bottleneck modules for for CT dataset. bn represents the bottleneck module.

Structural Models	SEN (%)	SPE (%)	ACC (%)	AUC (%)	PRE (%)	F1-Score (%)	Time (min)
WMR-DepthwiseNet-A: 3 (bn 3×3) + 5 (bn 5×5)	91.46	92.61	89.07	90.48	90.81	91.78	11.3
WMR-DepthwiseNet-B: 3 (bn3×3 ) + 6 (bn 5×5)	94.67	95.12	91.31	95.73	94.28	95.58	12.7
WMR-DepthwiseNet-C: 3 (bn 3×3) + 7 (bn 5×5)	95.41	96.92	94.55	95.82	95.14	96.86	12.5
WMR-DepthwiseNet-D: 3 (bn 3×3) + 8 (bn 5×5)	97.78	96.22	96.83	97.61	97.02	97.37	13.9
WMR-DepthwiseNet-D: 3 (bn 3×3) + 9 (bn 5×5)	94.1	93.7	95.1	94.9	95.1	94.7	14.8
WMR-DepthwiseNet-D: 3 (bn 3×3) + 10 (bn 5×5)	94.8	94.1	94.0	95.8	94.3	93.9	15.5

**Table 6:** Comparing the performance of our formulated WMR-DepthwiseNet with famous pretrained algorithms using the same CXR dataset. We only fine-tuned the last layer of the pretrained algorithms to match the number of classes.

Models	SEN (%)	SPE (%)	ACC (%)	AUC (%)	PRE (%)	F1 Score (%)	Time (min)
VGG-19	92.71	91.84	92.39	91.14	91.67	92.12	26.2
AlexNet	90.37	89.72	89.95	90.61	89.75	90.18	16.4
ResNet-50	95.73	96.18	94.23	95.76	93.92	94.86	25.9
EfficientNet	96.49	95.94	96.69	94.94	95.77	96.03	21.6
DenseNet-121	93.74	92.31	92.85	93.31	92.95	93.48	22.1
Inception-V3	91.88	90.75	91.31	90.96	90.21	91.66	19.7
MobileNet-V2	94.83	95.27	94.14	93.57	92.63	93.78	17.3
WMR-DepthwiseNet-D (Proposed)	98.46	97.99	98.63	98.72	98.69	98.92	15.6

**Table 7:** Comparing the performance of our formulated WMR-DepthwiseNet with famous pretrained algorithms using the same CT dataset. We only fine-tuned the last layer of the pretrained algorithms to match the number of classes.

Models	SEN (%)	SPE (%)	ACC (%)	AUC (%)	PRE (%)	F1 Score (%)	Time (min)
VGG-19	91.17	90.91	90.02	90.78	90.62	91.37	24.2
AlexNet	89.59	88.71	88.52	89.98	90.03	89.74	14.7
ResNet-50	94.82	95.62	93.23	93.45	91.87	92.17	23.4
EfficientNet	94.67	94.81	94.13	92.81	93.08	93.89	19.8
DenseNet-121	92.28	90.81	90.55	91.75	90.73	91.65	20.3
Inception-V3	90.03	89.24	90.88	89.32	89.13	90.78	17.6
MobileNet-V2	92.79	93.78	92.81	91.82	90.67	91.96	15.1
WMR-DepthwiseNet-D (Proposed)	97.78	96.22	96.83	97.61	97.02	97.37	13.9

**Table 8:** Evaluation performance of our proposed WMR-DepthwiseNet model in comparison with several COVID-19 image-based screening methods for both CXR and CT datasets.

Methods	SEN (%)	SPE (%)	ACC (%)
Chen et al. [11]	100	93.6	95.2
Barstugan et al. [40]	91.8	92.3	90.7
Wang et al. [12]	90.4	89.5	92.3
Li et al. [37]	90.0	96.0	92.3
Song et al. [42]	96.0	77.0	86.1
Shi et al. [41]	90.7	87.2	89.4
Wang et al. [33]	85.9	89.4	82.9
Jin et al. [53]	94.1	95.5	96.5
Xu et al. [52]	87.9	90.7	86.7
Jin et al. [54]	97.4	92.2	95.7
WMR-DepthwiseNet-D (CXR)	98.46	97.99	98.63
WMR-DepthwiseNet-D (CT)	97.78	96.22	96.83

**Table 9:** Comparison of our proposed WMR-DepthwiseNet with other selected state-of-the-art COVID-19 models using the same training data distribution for CXR dataset.

Model	SEN (%)	SPE (%)	ACC (%)	AUC (%)	PREC (%)	Time (min)
COVID-Net [33]	94.20	93.99	94.86	94.32	95.56	26.4
DeCoVNet [57]	97.21	97.68	97.78	97.21	97.41	22.8
Cov-Net [37]	97.92	96.28	97.67	96.27	97.65	23.7
DeepPneumonia [42]	90.72	91.20	90.78	90.06	91.80	25.8
WMR-DepthwiseNet-D (CXR)	98.46	97.99	98.63	98.72	98.69	15.6

**Table 10:** Comparison of our proposed WMR-DepthwiseNet with other selected state-of-the-art COVID-19 models using the same training data distribution for CT dataset.

Model	SEN (%)	SPE (%)	ACC (%)	AUC (%)	PREC (%)	Time (min)
COVID-Net [33]	92.37	92.54	93.81	92.65	93.16	24.9
DeCoVNet [57]	95.81	96.43	95.17	94.98	95.21	20.2
Cov-Net [37]	95.76	95.81	96.76	95.36	95.03	21.6
DeepPneumonia [42]	89.04	90.77	89.24	89.70	90.55	23.4
WMR-DepthwiseNet-D (CT)	97.78	96.22	96.83	97.61	97.02	13.9

**Table 11:** Evaluation of hyperparameter tuning on the overall performance of the proposed WMR-DepthwiseNet.

	CXR Dataset			CT Dataset		
Hyper-Parameter Tuning	SGD	Adam	RMSProp	SGD	Adam	RMSProp
	ACC (%)	ACC (%)	ACC (%)	ACC (%)	ACC (%)	ACC (%)
LR (0.1) + Dropout (0.25)	88.18	90.73	91.14	89.91	90.77	91.89
LR (0.1) + Dropout (0.50)	90.56	91.26	89.72	90.26	91.37	89.43
LR (0.1) + Dropout (0.75)	89.88	90.14	89.02	91.72	92.74	90.19
LR (0.01) + Dropout (0.25)	92.51	91.85	90.18	91.78	90.42	89.33
LR (0.01) + Dropout (0.50)	91.04	90.28	91.22	90.80	92.25	91.66
LR (0.01) + Dropout (0.75)	90.55	92.83	92.76	91.08	91.81	90.71
LR (0.001) + Dropout (0.25)	90.33	91.18	93.18	92.46	91.52	90.59
LR (0.001) + Dropout (0.50)	91.77	92.15	91.13	92.89	92.79	92.77
LR (0.001) + Dropout (0.75)	92.66	93.78	92.99	94.02	93.68	92.16
LR (0.0001) + Dropout (0.25)	94.38	94.13	93.23	94.38	94.17	94.89
LR (0.0001) + Dropout (0.50)	95.61	97.26	95.81	94.27	96.83	95.33
LR (0.0001) + Dropout (0.75)	94.79	95.76	93.98	93.16	94.72	94.79

**Table 12:** Dataset split for cross evaluation on CXR dataset.

Dataset	Category of Pneumonia	Data Count per Category	Selected No. of Data Category	Training Set	Validation Set	Test Set
Tabik et al. [58]	COVID-CXR	426	424	212	106	106
	NON-COVID-CXR	426	424	212	106	106
Rahman et al. [47]	COVID-CXR	3616	424	212	106	106
	NON-COVID-CXR	10,192	424	212	106	106
Total		14,656	1696	848	424	424

**Table 13:** Dataset split for cross evaluation on CT dataset.

Dataset	Category of Pneumonia	Data Count per Category	Selected No. of Data Category	Training Set	Validation Set	Test Set
Soares et al. [48]	COVID-CT	1252	424	212	106	106
	NON-COVID-CT	1230	424	212	106	106
Yang et al. [59]	COVID-CT	349	424	212	106	106
	NON-COVID-CT	463	424	212	106	106
Total		3294	1696	848	424	424

**Table 14:** Results of the cross-evaluation of the proposed model for CXR dataset.

Training Dataset	Test Dataset	ACC (%)	SEN (%)	SPE (%)
Rahman et al. [47]	Tabik et al. [58] (Train)	98.17	97.98	97.0
Rahman et al. [47]	Tabik et al. [58] (Test)	97.92	97.13	96.87
Rahman et al. [47]	Tabik et al. [58] (Train + Test)	98.01	97.88	97.09
Tabik et al. [58] (Train + Test)	Rahman et al. [47]	97.87	97.23	96.46

**Table 15:** Results of the cross-evaluation of the proposed model for CT dataset.

Train Dataset	Test Dataset	ACC (%)	SEN (%)	SPE (%)
Soares et al. [48]	Yang et al. [59] (Train/Val)	96.0	97.11	95.92
Soares et al. [48]	Yang et al. [59] (Test)	95.46	96.73	94.81
Soares et al. [48]	Yang et al. [59] (Train/Val + Test)	97.0	95.03	95.55
Yang et al. [59] (Train/Val + Test)	Soares et al. [48]	96.94	90.55	95.71

## Data Availability

In this study, we collected chest x-ray data of different pneumonia-related illnesses from three different open sources. We collected 3616 scans of COVID-19 CXR from the COVID-19 radiography database. We collected 3029 scans of bacterial pneumonia, 8851 scans of healthy patients, and 2983 scans of viral pneumonia from the Kaggle database of the Radiological Society of North America (RSNA). More so, we collected 74,999 scans of other pneumonia-related illnesses from the National Institute of Health (NIH). https://www.kaggle.com/tawsifurrahman/covid19-radiography-database (accessed on 1 June 2021). https://www.kaggle.com/c/rsna-pneumonia-detection-challenge/data (accessed on 2 June 2021). https://www.kaggle.com/nih-chest-xrays/data (accessed on 3 June 2021). https://github.com/ari-dasci/OD-covidgr (accessed on 2 June 2021). https://github.com/UCSD-AI4H/COVID-CT (accessed on 4 June 2021).
